# Male form of persistent Mullerian duct syndrome type I (hernia uteri inguinalis) presenting as an obstructed inguinal hernia: a case report

**DOI:** 10.1186/1752-1947-5-586

**Published:** 2011-12-20

**Authors:** Nishikant N Gujar, Ravikumar K Choudhari, Geeta R Choudhari, Nasheen M Bagali, Harish S Mane, Jilani S Awati, Vipin Balachandran

**Affiliations:** 1 Department of General Surgery, Al Ameen Medical College, Bijapur, Karnataka, India; 2 Department of Gynecology, Al Ameen Medical College, Bijapur, Karnataka, India; 3 Department of Pathology, Al Ameen Medical College, Bijapur, Karnataka, India; 4 Al Ameen Medical College, Bijapur, Karnataka, India

## Abstract

**Introduction:**

Persistent Mullerian duct syndrome is a rare form of male pseudo-hermaphroditism characterized by the presence of Mullerian duct structures in an otherwise phenotypically, as well as genotypically, normal man; only a few cases have been reported in the worldwide literature. We report the case of a 30-year-old man with unilateral cryptorchidism on the right side and a left-sided obstructed inguinal hernia containing a uterus and fallopian tube (that is, hernia uteri inguinalis; type I male form of persistent Mullerian duct syndrome) coincidentally detected during an operation for an obstructed left inguinal hernia.

**Case presentation:**

A 30-year-old South Indian man was admitted to our facility with a left-sided obstructed inguinal hernia of one day's duration. He had a 12-year history of inguinal swelling and an absence of the right testis since birth. Our patient had well developed masculine features. Local physical examination revealed a left-sided obstructed inguinal hernia with an absence of the right testis in the scrotum. Exploration of the inguinal canal revealed an indirect inguinal hernia containing omentum, the left corner of the uterus and a left fallopian tube. Extension of the incision revealed a well formed uterus, cervix and upper part of the vagina attached to the prostate by a thick fibrosed band. Total excision of the uterus, bilateral fallopian tubes and right testis was performed. A biopsy was taken from the left testis. The operation was completed by left inguinal herniorraphy. Histopathological examination of the hernial contents was consistent with that of a uterus and fallopian tubes without ovaries. Both testes were atrophied, with complete arrest of spermatogenesis. Post-operative karyotype analyses were negative for 46,XY and Barr bodies on buccal smear. A semen examination revealed azoospermia with a low serum testosterone level.

**Conclusions:**

In cases of unilateral or bilateral cryptorchidism associated with inguinal hernia, as in our patient's case, the possibility of persistent Mullerian duct syndrome should be kept in mind in order to prevent further complications such as infertility and malignant change. Hernia uteri inguinalis is the type I male form of persistent Mullerian duct syndrome, characterized by one descended testis and herniation of the ipsilateral corner of the uterus and fallopian tube into the inguinal canal.

## Introduction

Persistent Mullerian duct syndrome (PMDS) was first described by Nilson in 1939 [1]. Subsequently, approximately 150 cases have been reported in the literature [2].

PMDS is a rare form of male pseudo-hermaphroditism characterized by the presence of Mullerian duct structures in an otherwise phenotypically, as well as genotypically, normal man [3]. The exact cause of PMDS is not known, however it is thought to result from the defect of the synthesis or release of Mullerian inhibiting factor (MIF) or from a MIF receptor defect [2]. The persistence of a large uterus-like paramesonephric duct in a man is in itself clinically unusual, but when it forms a part of the contents of a hernial sac, it must be considered a rarity [4]. Hernia uteri inguinalis (male form of PMDS type I) is one of the rare causes of male pseudo-hermaphroditism [5].

This article describes the extremely rare finding of a left obstructed, indirect inguinal hernial sac in an adult man, namely a paramesonephric duct. We report the case of a 30-year-old man with unilateral cryptorchidism on the right side and a left obstructed inguinal hernia containing a uterus and fallopian tube (that is, hernia uteri inguinalis; type I male form of PMDS) coincidentally detected during an operation for an obstructed left inguinal hernia with right cryptorchidism.

## Case presentation

A 30-year-old South Indian man presented to our facility with a left-sided obstructed inguinal hernia of one-day duration with a history of left inguinal swelling from 12 years and absence of the right testis since birth. Our patient had been married for five years. He had no sexual dysfunction, but had primary sterility.

**Figure 1:**
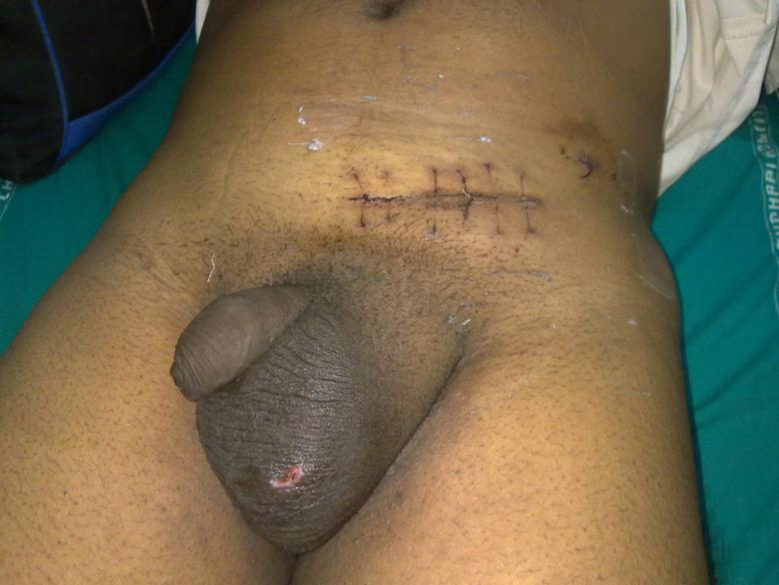
**Photograph showing well developed penis with poorly developed scrotum**.

He had no history of recurrent hematuria or family history of such disorders. A general physical examination revealed a man of muscular build with well developed secondary sexual characteristics. His urethra and penis were fully developed with a poorly developed right hemi-scrotum (Figure 1) and no palpable right testis in the scrotum or inguinal canal.

There was a non-reducible, tense, tender swelling measuring approximately 10 × 8 cm in the left inguinal region. A cough impulse was absent. Other examinations and routine investigations were normal. Our patient was prepared and taken for surgery.

**Figure 2:**
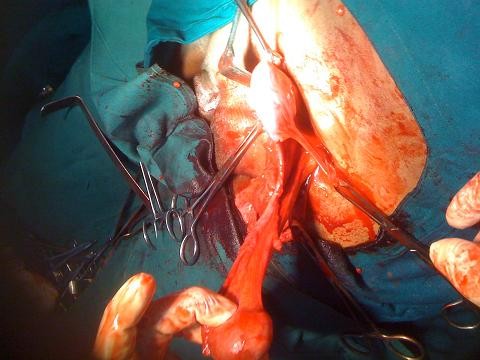
**Intra-operative photograph showing well formed uterus with fallopian tubes and left testes**.

**Figure 3:**
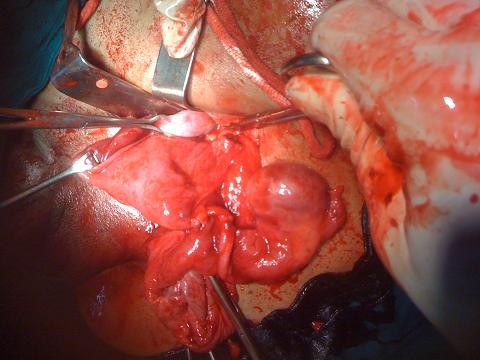
**Intra-operative photograph showing well formed uterus with fallopian tubes and right testis, which was embedded in the broad ligament**.

Exploration of the inguinal canal revealed an indirect inguinal hernia containing omentum, the left corner of the uterus and a left fallopian tube. Extension of the incision revealed a well formed uterus, cervix and the upper part of the vagina attached to the prostate by a thick fibrosed band with an atrophic right testis embedded in the broad ligament (Figures 2 and 3).

Total excision of the uterus with bilateral fallopian tubes and right testis was performed and the operation was completed with left inguinal herniorraphy. Though the left testis was atrophic, a biopsy was taken and it was left in place to sustain hormone secretion. Our patient recovered well post-operatively. To protect against the risk of malignancy in the testis, long-term follow-up was planned.

Grossly, the specimens removed were identified as a uterus with patent endometrial and endocervical linings and two fallopian tubes with no ovaries. The right testis measuring 1 × 1 × 1 cm, was atrophic and embedded in the right broad ligament. The left testicular biopsy specimen measured 0.5 × 0.5 × 0.5 cm.

**Figure 4:**
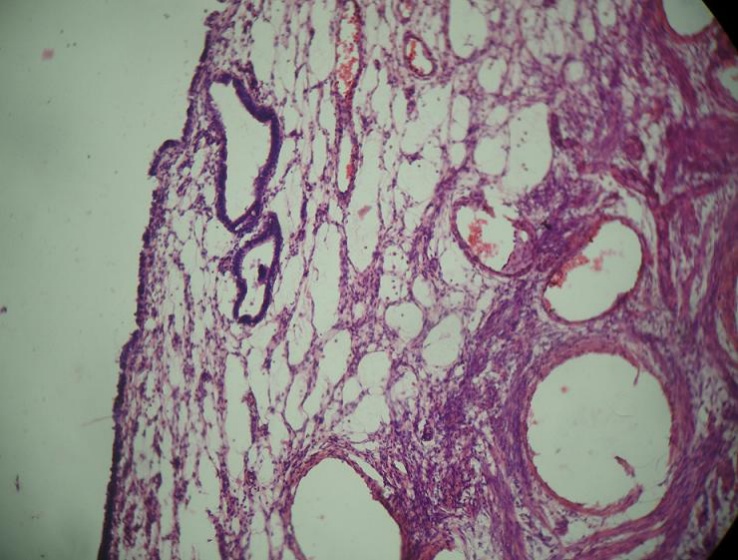
**Uterine muscular tissue with cavity lined by atrophied endometrial tissue**.

**Figure 5:**
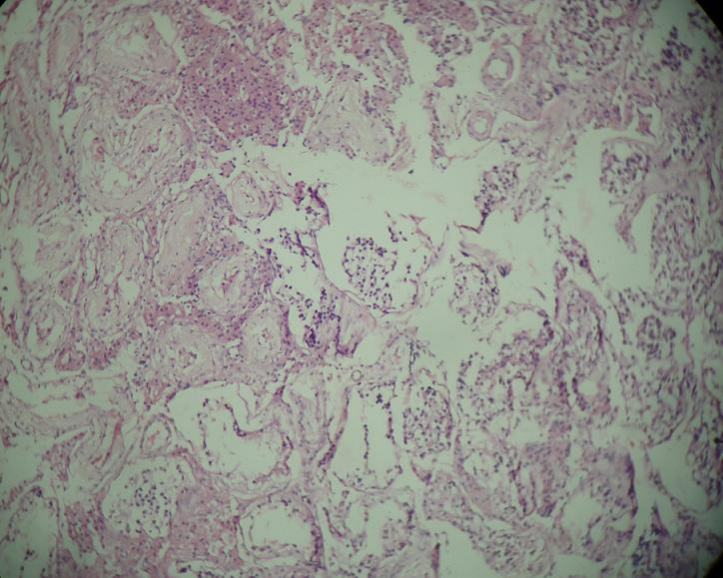
**Testis showing arrest of spermatogenesis and hyalinization**.

**Figure 6:**
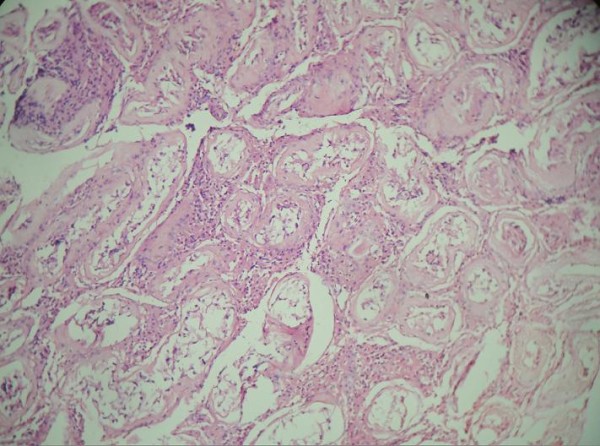
**Testicular atrophy with hyalinized semineferous tubules with complete arrest of maturation**.

**Figure 7:**
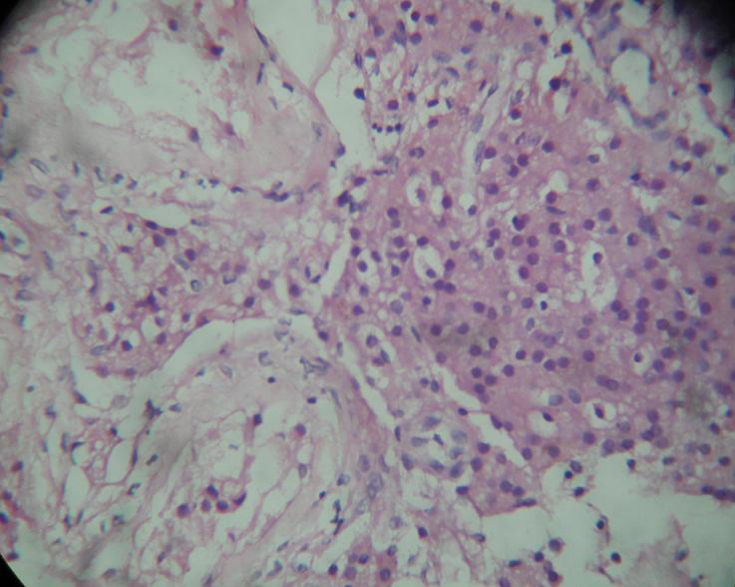
**Leydig cell hyperplasia**.

On histopathological examination, uterine muscular tissue with its cavity lined by atrophied endometrial tissue was seen (Figure 4). Sections from both fallopian tubes showed congestion and fibrosis. No ovarian tissue was seen. Sections from both testes showed atrophic semineferous tubules with hyalinization, Sertoli cells (Figures 5 and 6) and evidence of Leydig cell hyperplasia (Figure 7). No evidence of malignancy was seen in tissue samples from either testicle.

The final histopathological diagnosis of the hernial tissue was consistent with that of a uterus with fallopian tubes and no ovaries. Additionally, the testes were found to be atrophic with complete arrest of spermatogenesis.

Post-operative karyotype analyses were negative for 46,XY and Barr bodies on buccal smear. The post-operative serum laboratory test results are shown in Table 1. A semen examination revealed azoospermia.

**Table 1:** Post-operative serological test value results

Substance tested for	Value	Range	Comments
Testosterone	104.94 ng/dL	241 to 827 ng/dL	Low
Follicle-stimulating hormone	10 mIU/mL	1 to 14 mIU/mL	Normal
Leutenizing hormone	5.0 mIU/mL	0.7 to 7.4 mIU/mL	Normal
Thyroid-stimulating hormone	4 μU/mL	0.5 to 5 μU/mL	Normal
Adrenocorticotropic hormone	35 pg/mL	9 to 52 pg/mL	Normal
Prolactin	15 ng/mL	1 to 20 ng/mL	Normal
α-Fetoprotein	5 ng/mL	0 to 8.5 ng/mL	Normal
β-Human chorionic gonadotropin	3 IU/L	<5 IU/L	Normal
Lactate dehydrogenase	100 U/L	50 to 102 U/L	Normal

## Discussion

In true hermaphrodites, both ovarian and testicular tissue is present in one or both gonads [6]. In female pseudo-hermaphrodites, the gonads are ovaries, but male tendencies are seen in the organ of reproduction [7]. Conversely, male pseudo-hermaphroditism is a condition in which the gonads are testes but the internal genitalia are not completely virilized. Male intersex may present (1) with masculine external genitalia with fully developed uterus (as in our patient's case), (2) with purely feminine external genitalia, or (3) with external genitalia of equivocal sexuality [8].

It is possible for pseudo-hermaphroditism to be undetected until puberty [9]. PMDS is a rare form of internal male pseudo-hermaphroditism in which Mullerian duct derivatives are seen in men. It was first described by Nilson in 1939 [1]. Subsequently, approximately 150 cases have been reported. A familial association has been found in some cases [2].

The exact cause of PMDS is not known, however it is thought to result from a defect of the synthesis or release of MIF, or from defects in the MIF receptor. MIF is released by the Sertoli cells in fetal tissue from seven weeks of gestation onwards, and is responsible for the regression of the Mullerian duct in the male fetus. Defects in the MIF gene lead to the persistence of a uterus and fallopian tube in males. It is likely that remnant Mullerian structures lead to cryptorchidism by hindering the normal testicular descent mechanism [2].

Derivatives of the Mullerian duct, that is, the fallopian tubes, uterus and the upper part of the vagina, are present in a normal genotypically and phenotypically male individual. Patients with PMDS usually have normal development of external genitalia and secondary sexual characteristics [5].

The typical patient with PMDS has unilateral or bilateral cryptorchidism and is assigned to the male sex at birth without hesitation, as they have normal male genotypes and phenotypes [2]. Two anatomic variants of PMDS have been described: male and female. The male form is encountered in 80% to 90% of cases, characterized by unilateral cryptorchidism with contralateral inguinal hernia, and can be one of two types: the first type is hernia uteri inguinalis, which is characterized by one descended testis and herniation of the ipsilateral corner of uterus and fallopian tube into the inguinal canal. The second type is crossed testicular ectopia, which is characterized by herniation of both testes and the entire uterus with both fallopian tubes [5].

The female form, seen in 10% to 20% of cases, is characterized by bilateral cryptorchidism. The gonads are fixed within the pelvis, with the testes fixed within the round ligament in the ovarian position with respect to the uterus[5].

Clinically, the persistence of a uterus and fallopian tubes leads to either cryptorchidism or inguinal hernia depending on whether or not Mullerian derivatives can be mobilized during testicular descent [3]. If the uterus and fallopian tube are mobile, they may descend into the inguinal canal during testicular descent. However, if the Mullerian structures are relatively immobile testicular descent may be impeded [5,10,11].

PMDS is usually coincidently detected during surgical operation, as in our patient's case. However pre-operative ultrasonography, computerized tomography and MRI allow possible pre-operative diagnosis [3]. The prognosis depends upon the integrity of the testicular tissue and successful correction of cryptorchidism, which is often complicated by the close anatomical relationship between the vas deferens and the Mullerian derivatives [3].

The risk of malignancy in an ectopic testis in a case of PMDS is similar to that in a healthy male, with the incidence being 15%. There have been case reports of embryonal carcinoma, seminoma, yolk sac tumor and teratoma in patients with PMDS, whereas tumors of the Mullerian duct derivatives are very rare [2,5]. Infertility is common, with an absence of spermatozoa observed during semen analysis [5].

The main therapeutic considerations are the potential for fertility and prevention of malignant change. Surgical management is geared towards preserving fertility, and orchiopexy, which is performed to retrieve the testis and position it in the scrotum, should be performed early to maintain fertility with care taken not to damage the vas deferens during the operation. The uterus is usually removed and attempts are made to dissect away Mullerian tissue from the vas deferens [2,3].

Additionally, routine orchiectomy is not recommended as the capacity of the virilization must be maintained and is only indicated for testes that cannot be mobilized to a palpable position [2].

The risk of developing malignancy is greater in an abdominal localization than in an inguinal testis [3]. Therefore, in our patient's case, the right atrophic testis was removed. Despite the risk of malignancy and no chance of fertility, the left testis was protected to maintain virilization.

## Conclusions

PMDS is a rare form of male pseudo-hermaphroditism characterized by the presence of Mullerian duct structures in an otherwise phenotypically, as well as genotypically, normal man. The patient with PMDS has unilateral or bilateral cryptorchidism and is usually assigned to the male sex at birth without hesitation. Since patients are phenotypically male, the diagnosis is usually not suspected until surgery is performed for cryptorchidism or hernia repair.

Hernia uteri inguinalis is type I of the male form of PMDS, characterized by one descended testis and the herniation of the ipsilateral corner of the uterus and fallopian tube into the inguinal canal. In order to prevent further complications such as infertility and malignant change, the surgeon should be aware of PMDS while dealing with patients who present with unilateral or bilateral cryptorchidism.

In summary, in cases of unilateral or bilateral cryptorchidism associated with hernia, as in our patient's case, the possibility of PMDS should be kept in mind.

## Consent

Written informed consent was obtained from the patient for publication of this case report and any accompanying images. A copy of the written consent is available for review by the Editor-in-Chief of this journal.

## Competing interests

The authors declare that they have no competing interests.

## Authors' contributions

RC, NG and GC performed the surgery. NG and RC analyzed and interpreted clinical data from our patient and were also major contributors in writing the manuscript. NB and HM conducted the histopathological study. JA and VB assisted in the overall study. All authors have read and approved the final manuscript.
